# Solubility and Bioavailability Enhancement of Oridonin: A Review

**DOI:** 10.3390/molecules25020332

**Published:** 2020-01-14

**Authors:** Yuanyuan Zhang, Shaohua Wang, Mengmeng Dai, Jijuan Nai, Liqiao Zhu, Huagang Sheng

**Affiliations:** College of pharmacy, Shandong University of Traditional Chinese Medicine, Jinan 250355, China; zhangyuan13zyy@163.com (Y.Z.); wang642020531@163.com (S.W.); m17862968199@163.com (M.D.); Ne19863675828@163.com (J.N.)

**Keywords:** Oridonin, structural modification, formulations, solubility, bioavailability, biological activity

## Abstract

Oridonin (ORI), an ent-kaurene tetracyclic diterpenoid compound, is isolated from Chinese herb *Rabdosia rubescens* with various biological and pharmacological activities including anti-tumor, anti-microbial and anti-inflammatory effects. However, the clinical application of ORI is limited due to its low solubility and poor bioavailability. In order to overcome these shortcomings, many strategies have been explored such as structural modification, new dosage form, etc. This review provides a detailed discussion on the research progress to increase the solubility and bioavailability of ORI.

## 1. Introduction

**Figure 1:**
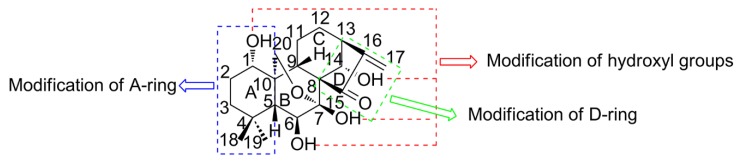
Molecular structure and typical modification sites of Oridonin (ORI).

“Donglingcao” is the dry aerial part of Labtea *Rabdosia rubescens* (Hemsl.) Hara. The 2015 edition of the Chinese Pharmacopoeia records its efficacy of clearing away heat and detoxifying, promoting blood circulation and relieving pain. It is commonly used in the treatment of acute and chronic pharyngitis, bronchitis, tonsillitis and insect bites [1,2]. The main chemical component is the diterpenoid compound, and the ent-kaurene tetracyclic diterpenoid compound represented by Oridonin (ORI, C_20_H_28_O_6_, Figure 1) exhibits strong antitumor activity [3,4]. ORI also has anti-bacterial [5], anti-inflammation [6], anti-oxidation [7], depressurization [8], anti-inflammation [9,10,11], immune-modulating [12] and analgesia [13] effects (Table 1). In particular, ORI demonstrates a significant inhibitory effect on the proliferation of more than 20 human cancer cell lines, such as common esophageal cancer [14], lung cancer [15], liver cancer [16], prostate cancer [17], breast cancer [18], and colorectal cancer [19], which is attributed to its specific molecular structure. The α-methylene cyclopentanone (enone) on the D-ring is the essential structure of the anti-cancer activity, which can be impaired by ring splitting or methylene saturation [20]. Besides, the hydrogen bond between the 6-hydroxy group and the 15-carbonyl group increases the electrophilicity of C(17), which further improves the affinity to electrophilic enzymes in tumor cells. The anti-cancer activity can be enhanced by the esterification of 14-hydroxyl group whose nucleophilic property is amplified by the parallel 7-hydroxyl group; the antitumor activity decreases without the 7-hydroxyl group [21].

Despite the wide spectra of pharmacological activities, the application of ORI is limited due to its hydrophobicity. It has a solubility of 0.75 mg/mL and the log *p* = 1.66, leading to disappointing bioavailability [22]. Moreover, the rapid plasma clearance of ORI, the first-pass effect after oral administration, and the lack of proper dosage forms for intravenous injection hinder pharmacodynamics research and clinical development of ORI [23]. According to a previous report, when orally administered to rats (20, 40 and 80 mg/kg), the absolute bioavailability of ORI is 4.32%, 4.58% and 10.80% in a dose-dependent manner [24]. At present, the clinical use of *Rabdosia rubescens* is limited to tablet and drop pills forms; it can detoxify and relief pain [25] or be combined with other chemical drugs to enhance the anti-cancer effect [26].

To alleviate the deficiency of ORI applications, much effort has been made on the improvement of the solubility and bioavailability of ORI. Among the various strategies, structural modification shows remarkable efficacy in boosting solubility and biological activity [11], and the development of new technologies as well as dosage forms inspires the regulation of ORI delivery via pharmaceutics method [27]. These achievements lay a stepping stone and disclose the great potential future applications of ORI. In this review, we summarize the strategies to improve the solubility and bioavailability of ORI by structural modifications and build-up of drug delivery systems over recent years.

## 2. Strategies for Structural Modification

Structural modification has been broadly processed to optimize the solubility and membrane permeability of ORI, which contributes to enhanced bioavailability. Generally, the main modification sites include the A-, B-, and D-ring and the corresponding hydroxyl or methylene groups (Figure 1) [28,29]. Here, several crucial modification strategies will be introduced.

### 2.1. Thiazolation of ORI

**Figure 2:**
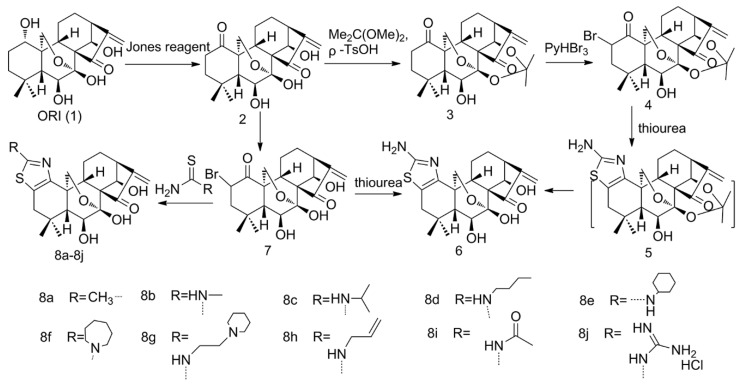
Routes for introduction of thiazole rings at the C(**1**) and C(**2**) sites on ORI.

Modification of the thiazole ring has been certified effective in improving solubility and biological activity [30]. After introducing the thiazole ring into the C(**1**) and C(**2**) on A-ring, this structure could interact with the acid and forms salt so as to improve the water solubility with the nitrogen atoms bound to the core scaffold of the ORI; meanwhile, a biologically active enone moiety was kept intact (Figure 2). It has been reported that the solubility of hydrochloride of compound **8b**, **8h** was 62 and 32 times that of bulk ORI (1.29 mg/mL) respectively. In addition, compounds **8a**–**8e** and **8g**, **8h** showed a significant anti-proliferation effect on breast, pancreatic and prostate cancer cells with IC_50_ (50% inhibiting concentration) within the range 0.1–1 μmol/L, as well as the multidrug resistance cells ER(+) MCF-7 [31]. Claire et al. used the alamar Blue Assay to measure the proliferation activity of human and rat hepatic stellate cell (HSC) lines, and in vitro studies showed that compound 8h had a significant inhibitory effect on hepatic fibrosis via suppression of the NF-κB pathway, which could significantly inhibit the proliferation of LX-2 and HSC-T6 cells and induce their apoptosis [32].

### 2.2. Glycosylation of ORI

**Figure 3:**
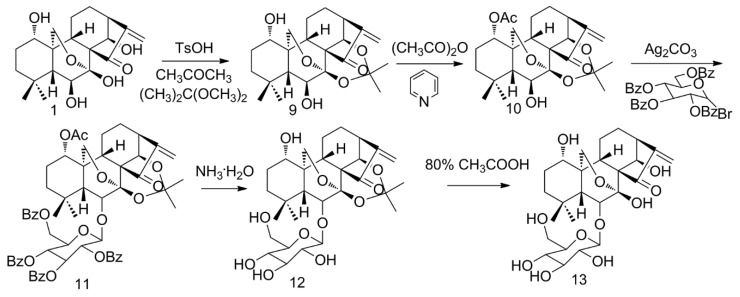
Synthesis of ORI-6-*O*-β-d-glucopyranoside.

Glycosylation is regarded as an ideal modification strategy for ORI. For one thing, the derivatives of saccharides regulate various biological activities and indicate precious value in medical treatment. Secondly, glycosylation improves solubility and works as the ligand for targeted delivery in certain conditions [33]. After protecting hydroxyl groups at C(**1**), C(**7**) and C(**14**), ORI reacted with α-d-Glucopyranosyl bromide tetrabenzoate via a Koenigs–Knorr reaction and the typical glycosylation of ORI (ORI-6-*O*-β-d-glucopyranoside) was synthesized after deprotection (Figure 3) [34]. Generally, glucose transporter 1 is overexpressed in certain tumor cells [35], which require more glucose to maintain growth and proliferation than normal cells. It is expected that the glycosylation of ORI might increase its cancer cellular uptake, so further activity studies are worthwhile.

### 2.3. PEGylation and Esterification of ORI

**Figure 4:**
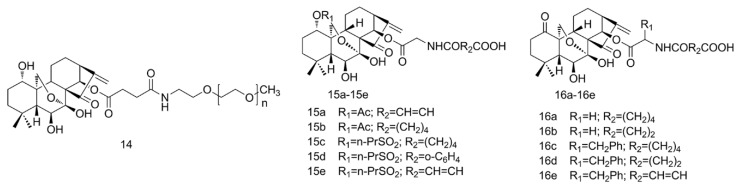
PEGylation and esterification of ORI.

Polyethylene glycol (PEG) is a widely used polymer in drug conjugation due to its unique physicochemical properties including good solubility, being degradable, low cost, good biocompatibility, steric repulsion [36]. With PEGylation, the hydrophilicity of the drugs can be effectively enhanced and the circulation time of drugs are prolonged by avoiding reticuloendothelial system (RES) clearance [37]. PEGylating the carbonylated ORI by various MeO-PEG-NH_2_ conjugations at the C(**14**) position (PEG-SA-ORI, Figure 4, **14**), the drug loading efficiency and in vitro release profiles could be adjusted by altering the molecular weight (Mw) of PEG chains. As a result, the loaded-ORI content and solubility increased as Mw decreased. Compared with the bulk ORI, the solubility of the conjugates increased by 4.68–99.2 times, and the pharmacokinetics study showed that the area under [the plasma concentration time) curve (AUC) of the preferred PEG_20kDa_-SA-ORI was about 2.1-times higher, suggesting that PEGylation was a promising approach to achieve better bioavailability of ORI [38].

Apart from PEGylation, some other esterification methods were performed to modulate ORI properties. For instance, novel 1-O- and 14-O-ORI (Figure 4, **15a**–**15e**,**16a**–**16e**) derivatives were synthesized by introducing water-soluble chains through the attachment of ester groups, and the solubility of most compounds was improved (>50 mg/mL). The cytotoxicity of compound **15a**, **16e** was almost 40-times that of ORI in BEL-7402 cells, and the in vivo H22 or B16 tumor weight was up to 44.3% smaller than that of ORI in mice [39].

### 2.4. Amino Acid Modification of ORI

**Figure 5:**
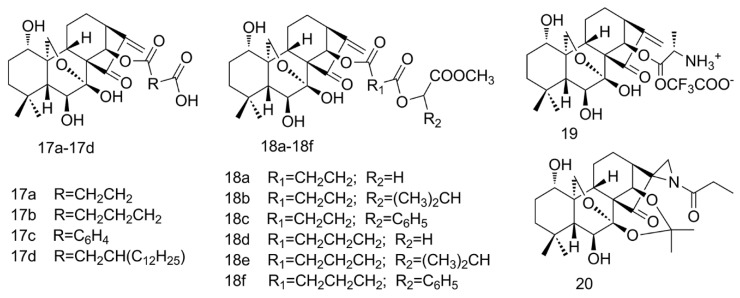
Amino acid modification and aziridination of ORI.

Amino acid is an important nutrient for metabolism in humans [40]. With both a basic amino group and an acidic carboxyl group, amino acid modification can be performed flexibly to fit the pH environment, improve the solubility or cell permeability, increase the targeting capability, or achieve sustained release profiles with proper dosage forms [41]. A series of 14-*O*-derivatives were synthesized with condensed anhydrides at 14-hydroxyl group followed by esterification with amino acid (Figure 5, **17a**–**17d**, **18a**–**18f**). It was found that the cell uptake of the derivative increased. Cytotoxicity could be amplified by controlling the length and decoration of the lipophilic side chain: The longest, **17d,** showed the strongest toxicity, and the cytotoxicity of compounds **18e** and **17c** of the aryl substituent was seven-times higher than that of the bulk ORI in the BGC-7901 cell. The in vivo tumor inhibition rate of **17c** was 16.9% higher than that of ORI and was comparable to that of cyclophosphamide (59.6% vs. 59.1%), despite the mechanism not being clear [42].

There are some other forms of ORI, like prodrugs, which improve their solubility and pharmacokinetic properties for long-term effects [43]. Sun et al. invented a method to prepare an aqueous soluble prodrug of ORI from amino acids [44], l-alanine-(14-oridonin) ester trifluoroacetate (HAO472) (Figure 5, **19**), which had good chemical stability and could be better used in clinical treatments with low toxicity and residue of crystallization solvent. HAO472 has advanced into Phase I human clinical trials for the treatment of acute myelogenous leukemia in China by Jiangsu Hengrui Medicine Co. Ltd.

### 2.5. Aziridination of ORI

Covalent drugs are the only class of drugs that can completely shut down and silence the protein activity that causes disease. In recent years, with the advent of targeted covalent inhibitors, the notion of exploring new generations of covalent drugs has resurged. Covalent drugs can react directly with targets to form covalent bonds, resulting in corresponding biological activity [45]. It can achieve its unique therapeutic advantages with strong and long-lasting effects and high ligand efficiency [45,46]. The enone system in the D-ring of natural covalent drug ORI, a classic covalent warhead, is identified as a Michael-acceptor necessary for biological activity, which reacts with various thiol groups and residues of small molecules (e.g., GSH) and target proteins (e.g., thioredoxin reductase) to afford irreversible inhibition, leading to potential non-targeting effects and clinical safety issues [47]. Removal of the enone fragment could cause the loss of ORI activity [19].

In order to reduce the toxicity of ORI and increase its safety, Ding et al. utilized a mild and efficient Rh_2_(esp)^2^-catalyzed reaction to regio- and stereo-pecifically constructed new aziridinated oridonin analogues, replacing the overly reactive enone at the same site on D‑ring [48]. In addition, aziridines are milder in chemical reactions and have better water solubility. The antiproliferative effect of ORI D‑ring aziridinated analogue (YD0514) (Figure 5, **20**) on the highly aggressive triple-negative breast cancer cell line MDA-MB231 was significantly enhanced with an IC_50_ value of 8.32 μM, compared with ORI (IC_50_ value of 29.4 μM), and it was less toxic to normal breast epithelial cells and induced the apoptosis of breast cancer cells in a dose-dependent manner [48]. In all, the various modification strategies provide an inspiring starting point to improve the application potential of ORI.

## 3. Strategizes for Pharmaceutical Formulations

Besides the properties of the drug itself, the full extent of drug activity is largely determined by the dosage form, which influences the absorption, release profile, stability, in-site accumulation, etc. In recent years, flourishing technologies like microparticles, solid dispersions, inclusions [49], liposomes and nanotechnology [50] have laid a solid foundation for the development of new dosage forms and the improvement of the quality of formulations. With appropriate delivery systems, it is possible to improve solubility, change the in vivo process, and enhance the bioavailability of ORI.

### 3.1. Cyclodextrin Inclusion Complexes

Cyclodextrin is a cyclic oligosaccharide compound composed of a glycoside-bound glucose unit. Its stereo configuration is a cylindrical structure with the core for the drug embedded in it to form an inclusion complex. Both ends and the outside surface of the cyclodextrin are hydrophilic, while the inner space is hydrophobic [51]. With ^1^H-NMR spectroscopy and two-dimensional rotating frame overhauser effect spectroscopy, it was confirmed that the A ring of ORI derivatives was anchored into the hydrophobic cavity from the wider edge of β-CD, while the rest was exposed outside of the cavity by preparing three ORI derivatives of β-cyclodextrin (β-CD) inclusion compounds [52].

Due to this special structure, cyclodextrin is often used as a carrier in pharmaceutics to increase drug solubility and stability, solidify liquid drugs, and reduce irritation, etc. [53]. An ORI-loaded inclusion complex prepared using 2-hydroxypropyl-β-cyclodextrin (HP-β-CD) by freeze-drying method, and the solubility of ORI-HP-β-CD inclusion was 27-times that of ORI (55.45 mM vs. 2.062 mM). In vivo tissue distribution studies showed a potential treatment for lung-targeting: The inclusion distribution increased in the lung while it decreased in the heart, spleen, and kidney compared with free ORI, which might be due to the its specific binding to substances in the lung [54].

### 3.2. Microparticles

The microparticle drug delivery system has been a research hotspot on pharmaceutical preparations in recent years. With encapsulation in microparticles, the solubility and bioavailability of poorly soluble drugs can be significantly enhanced. In addition, the dispersion system of particles in different sizes usually shows unique biodistribution, indicating its potential application in targeted delivery [55].

The large pore structure and small aerodynamic diameter of the polylactic acid-glycolic acid (PLGA) large porous microparticle (LPMP) prevented the ORI from phagocytosis of alveolar macrophages. The ORI-loaded porous PLGA microspheres prepared with electrospraying had high lung deposition after pulmonary administration; thus, this could be used as a dry powder inhaler for local treatment of lung cancer by inhibiting blood vessel generation and enhancing tumor apoptosis [27]. At present, the anti-cancer effect of some LPMPs is comparable to first-line clinical anti-cancer drugs, and regarded as an ideal delivery system for the treatment of primary non-small cell lung cancer [56].

Another new photosensitive ORI-loaded microsphere was developed by introducing porphyrin ring to the chitosan (CS) as a side group. ORI was released from CS microspheres in sustained profiles when exposed to light, with the fatality rate of 82.74% on MCF-7 cells at 100 μmol/mL, significantly higher than that in the dark (68.50%) [57].

Folate receptors (FR) are overexpressed on the surface of many cancer cells with high affinity to folic acid. With surface-coated folic acid-polyvinyl alcohol ester on liposome microbubbles, the targeting ultrasonic reaction delivery system F-LMB-ORI was further developed, where ORI released from the microbubbles flowing through the targeted area under high-intensity and led to higher ORI absorption. The in vitro inhibition rate of HepG2 cells was as high as 94.0%. The tumor inhibition rate was 89.4% in mice at a dose of 1.5 × 10^−2^ g/kg/day, which was significantly higher than that of the uncoated group (71.5%) [58].

### 3.3. Nanocrystals

Nanosuspension is a submicron colloidal dispersion without any carrier tools, which consists of drug crystal particles and a stabilizer, with up to 100% encapsulation efficiency (EE) [59]. Due to its small particle size, large specific surface area and high drug loading (DL), nanosuspension can efficiently deliver drugs and obtain higher intracellular concentrations, thus achieving greater efficacy [60]. Owing to the high melting point of crystal form, nanosuspension is favored for sustained release with low toxicity and high safety [61], and can be further manufactured into various drug delivery systems according to clinical requirements [62,63].

The preparation methods of nanosuspension mainly include top-down and bottom-up, or the combination of the two. The working principle of top-down preparation is to mechanically shear the drug crystal into nanometer-sized drug particles, of which media milling and high-pressure homogenization (HPH) are the most representative technologies [64,65]. Bottom-up preparation is based on the precipitation process of drug particles from their supersaturated solution, including antisolvent precipitation method, supercritical fluid precipitation and solvent removal precipitation method. Particularly, HPH is most widely used in nanosuspension technology development [66]. In recent years, the carrier-free nanosuspensions has been increasingly utilized to solve the problem of poor solubility and low bioavailability of ORI.

An ORI nanosuspension (ORI-N) prepared by HPH method has good stability when the ratio of drug to stabilizer is 1:5. In this case, ORI-N was uniformly dispersed in terms of solubility and the dissolution rate improved [67]. Altering the pressure and cycle numbers affected the ORI-N particle sizes obviously: Nanosuspension A was 103.3 ± 1.5 nm, while B was 897.2 ± 14.2 nm. It was found that the dissolution was largely affected by particle size: The accumulative dissolution of A in 10 min reached 99.9%, significantly higher than that of B (75.2% in 30 min), and A demonstrated higher heart and kidney accumulation compared with ORI solution. The plasma clearance rate of B was delayed, AUC was about twice that of the ORI solution and the MRI extended from 1.89 h to 11.78 h. The uptake ratio in the liver and spleen were 40.12% and 30.22%, respectively, which were significantly higher than that of nanosuspension A and ORI solution, providing a strong basis for optimizing the particle size for intravenous administration [68].

ORI-N has been widely investigated in both in vitro and in vivo tumor therapeutics. It has been reported that the inhibition effect of ORI-N on the K562 cells and the tumor volume of S−180 tumor-bearing mice was significantly higher than that of free ORI [69]. A similar effect was observed in PC−3 cells with ORI-N. [70]. Another study showed that ORI-N effectively induced the apoptosis of SMMC−7721 cells in the G2/M phase with cytotoxicity 5.88-times that of the ORI solution. In vivo studies showed that the repression of tumor was observed from the ninth day after ORI-N administration (20 mg/mL) [71]. This mechanism of cell cycle disruption has also been utilized in apoptosis of MCF-7 cells. With ORI-N administration, downregulation of Bcl-2 and the high expression of Bax were more significant compared with free ORI in time- and dose-dependent, which indicated induced apoptosis to a larger degree [72]. A greater reduction of the ratio of Bcl-2/Bax and activation of caspase-3 could also be realized with ORI-N in human pancreatic cancer PANC-1 cell line, as well as stronger cytotoxicity [73].

It is a novel trend to combine nanosuspension with cyclodextrin complexation technologies. The surfactant in the nanosuspension can stabilize the drug crystals, compensating for the shortcomings of drug outflow when using cyclodextrin inclusion complexes (CICs) alone. Meanwhile, the HP-β-CD and poloxamer enhance and increase the dissolution and the permeability of the drug in the intestine [74,75]. The bioavailability of ORI-loaded nanosuspension formulation of cyclodextrin inclusion complex prepared by solvent evaporation and wet media milling method soared to 213.99%. The surfactant poloxamer and CICs enhanced the apparent solubility of ORI by 11.2 times (from 0.524 mg/mL to 5.87 mg/mL) [76]. Table 2 lists these studies about ORI nanosuspension.

### 3.4. Nanoparticles

#### 3.4.1. Polymer Nanoparticles

Polymer nanoparticles are favored due to the chemical modification diversity, probable long-term circulation and targeting delivery in the body [77]. At present, various polymers have been investigated to build up ORI delivery systems. For instance, Poly (D, l-lactic acid) (PLA) is one of the common polymers with good biocompatibility, degradability and low toxicity. ORI-loaded PLA nanoparticles (ORI-PLA-NP) were prepared by a modified spontaneous emulsion solvent diffusion method, which increased the solubility of ORI and prolonged the blood circulation. High concentrations of ORI were observed in the liver, lung and spleen, but less so in the heart and kidney. However, ORI-PLA-NP has the disadvantage of small DL and can still be improved [78].

Hydrophilic poly-ethylene oxide (PEO) prevented NPs from clearance by the RES, prolonged the systemic circulation, and enhanced drug accumulation in tumor cells [79]. Feng et al. prepared an ORI-loaded poly (ε-caprolactam) poly (ethylene oxide) poly (ε-caprolactam) (PCL-PEO-PCL) copolymer nanoparticles (ORI-PCL-PEO-PCL-NPs) by interfacial deposition method. The tumor volume and weight were significantly smaller compared with the blank group and had a survival rate of 169.6% [80]. Another study successfully determined the pharmacokinetic parameters of ORI-PCL-PEO-PCL-NP in rabbit plasma by HPLC method, where the apparent volume of distribution was approximately twice that of the ORI solution [81].

Compared with passive targeting, active targeting utilizes the ligand-modified carrier as a “missile” to deliver the drug to the target sites and demonstrates better in-site drug accumulation and less toxicity. Modified with peptide Arg-Gly-Asp (RGD), ORI-PLA-RGD-NPs was prepared and achieved lower burst release and engulfment of phagocytic cells with much higher ORI accumulation in the tumor, liver and spleen. The pharmacodynamics study showed that the tumor volume and weight of ORI-PLA-RGD-NPs group were about half that of the ORI-PLA-NPs group, and the average survival of H22 tumor-bearing mice was about 14 days longer [82].

The asialoglycoprotein receptor (ASGP-R) is overexpressed on the surface of hepatoma cells, on which the membrane-bound active site can specifically bind to CS with reactive functional groups, high charge and biodegradability [83]. Based on this property, galactosylated chitosan (GC) was synthesized and coated on ORI-loaded nanoparticles (ORI-GC-NP) to cure hepatocellular carcinoma. In vitro drug release exhibited pH-dependence due to the CS-NH_2_ protonation in the acidic environment, resulting in the infiltration and release of ORI-GC-NP in acidic cancer cell environments. ORI-GC-NP mainly aggregated in the liver, and the AUC value of ORI-GC-NP in the liver was 6.4-times higher than the ORI solution, which confirmed its significant liver-targeting effect and suggesting it may be a promising ORI delivery system [84].

Another example is ORI-loaded galactosylated bovine serum albumin nanoparticle (ORI-GB-NP). The lactobionic acid was coupled to bovine serum albumin (BSA) by amidation reaction to synthesize GB. This was followed by the introduction of galactosamine on the free aldehyde groups for end-blocking. Then, the compound was prepared into nanodrugs by the desolvation technique [85]. ORI-GB-NP showed specific recognition and binding to ASGP-R, prolonged blood circulation and less distribution in heart, lung and kidney compared with ORI-BSA-NP, and the plasma concentration was more than twice that of the ORI solution [86].

**Figure 6:**
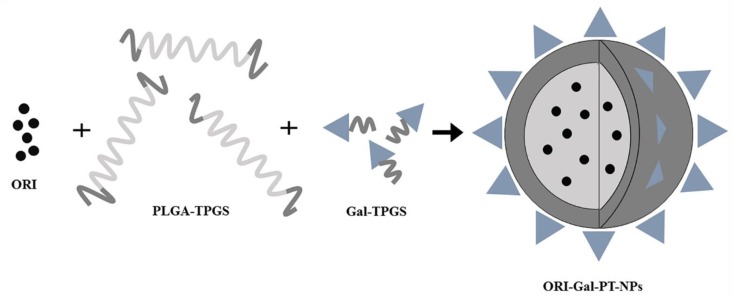
Schematic diagram of the preparation of ORI-Gal-PT-NPs.

Wang et al. synthesized galactosylated polymer nanoparticles modified by a double-ended D-α‑Tocopheryl polyethylene glycol succinate (TPGS) (ORI-Gal-PT-NPs) (Figure 6) [87]. TPGS could inhibit the expression of efflux pump *P*-glycoprotein and synergistically induce apoptosis with ORI [88,89]. ORI-loaded NPs accumulated in the tumor tissue through the enhanced permeability and retention effect. The galactose-specific receptor-mediated endocytosis increased the absorption of ORI-Gal-PT-NPs on HepG2 cells, and the apoptosis rate increased by 4−5.6% compared with the ORI solution [87]. Table 3 shows all the ORI polymer nanoparticles.

#### 3.4.2. Lipid Nanoparticles

##### Solid Lipid Nanoparticles

**Figure 7:**
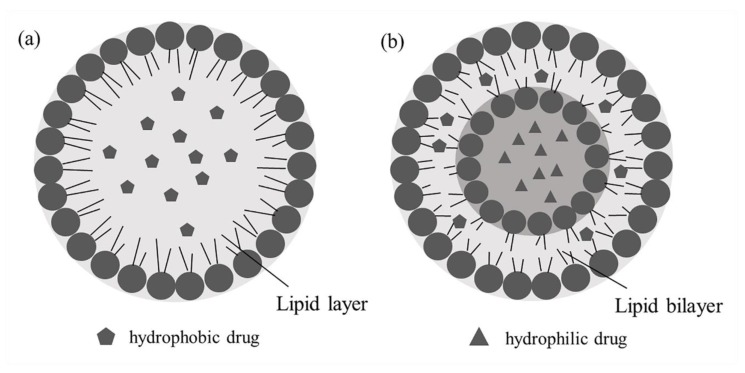
Structural comparison of lipid nanoparticles (**a**) and liposomes (**b**).

Solid lipid nanoparticles (SLNs), a new type of nanoparticle delivery system [90], emerged in the 1990s, bringing the advantages of high physical stability, low drug leakage, good sustained release profile and high bioavailability (Figure 7a) [91]. SLNs can also reduce the potential toxicity of drugs using biocompatible and high melting point lipids [92]. Therefore, SLNs are regarded as a promising carrier for drug delivery.

Zhang et al. used stearic acid, soya bean lecithin and pluronic F68 as the carrier materials to prepare the ORI-loaded SLNs. ORI was dispersed in SLNs in amorphous form with the EE over 40% [93]. It was reported that ORI-SLNs induced cell cycle arrest and the apoptosis of breast cancer cells MCF-7 more significantly than free ORI (*p* < 0.01), and the relative uptake rates (Re) in liver and spleen reached 41.25% and 31.44%, indicating that SLNs are an effective carrier for enhancing the anti-cancer activity of ORI [94].

##### Nanostructured Lipid Carriers

The nanostructured lipid carrier (NLC) is a second-generation lipid nanoparticle which combines the advantages of nanoparticles and liposomes similar to SLCs [95]. Generally, NLCs are in a porous matrix structure composed of liquid lipids, solid lipids and surfactants [96]. The defects of the nanoparticle lattice reduce the amount of drug discharged from the carrier material and are beneficial to the sustained release [97]. In addition, as the solubility in the liquid lipid is larger than the solid lipid, it may be a reasonable strategy to improve the DL by controlling the ratio of liquid lipids [98,99]. After oral administration of NLCs, lipids are digested in the gastrointestinal (GI) tract, increasing the residence time and absorption of the oral anti-cancer drugs [100]. Safety is regarded as one of the most important properties of NLCs, as its low glass transition temperature, low tendency for unpredictable gelation, low toxicity to normal tissues and reduced GI irritation attest [101,102]. 

With monostearate used as solid lipid and octanoic acid/capric triglycerides (CT) as liquid lipid, Dai et al. prepared a novel ORI drug, using nanostructured lipid as the carrier, with good physical stability. X-ray diffraction results showed the crystal form of this lipid nanoparticle. Despite the average particle size decreasing favorably to 200 nm, the core space of the nanoparticle was expanded to accommodate drug molecules, leading to higher EE (74.25%) and DL (3.55%) than NLCs without CT (40.52% and 1.97%, respectively) [103]. It was reported that the distribution of ORI-loaded NLCs in the liver was 8.6-times that of the ORI solution after intravenous administration at a dose of 25 mg/kg in Kunming germline mice [104]. To improve the pharmacokinetics behaviors of ORI-loaded NLCs, PEG was coated on the surface for long-circulation [105]. The MRT of ORI-PEG-NLCs was extended to 6.209 h, compared with 3.004 h of ORI-NLCs, with AUC increasing by 1.3 times.

Another way to optimize the absorption of ORI is active-targeting companied with avoiding hydrolysis of NLCs by digestive enzymes. Zhou et al. reported biotin-decorated ORI-loaded NLCs (Bio-ORI-NLCs), which increased oral relative bioavailability by 171.01% and effective intestinal permeability by three times via the interaction of biotin and intestinal receptors. Besides, the lipids could be reconstituted into new drug carriers (e.g., micelles) under digestion with GI lipase, by which the ORI was effectively protected from degradation before absorption. However, an in vitro study showed that ORI was strongly metabolized by liver enzymes in the first-pass metabolism (phase II), suggesting this system still requires further improvement for oral administration of ORI [106].

Recently, lipid-polymer hybrid nanoparticles (LPNs) attracted research interest by unitizing the advantages such as controlling drug release, avoiding drug degradation or leakage and good targeting. With specific modifications, this functional system demonstrated favorable potential for oral ORI administration. For example, surface modification of ORI-load LPNs used the specific adhesion property of wheat germ agglutinin (WGA), which increased the biofilm adhesion of drugs and reduced the clearance of LPNs in the GI tract [107]. By interacting with mucin, WGA-LPNs overcame mucosal barriers, and improved Re in the Caco−2 model. An in vivo study showed that the bioavailability of WGA-LPNs and LPNs increased 9.09 times and 1.96 times compared with ORI suspension, respectively [108].

### 3.5. Liposomes

Liposomes are microcapsules (Figure 7b) that can encapsulate both hydrophilic and hydrophobic drugs [109]. Commonly designed for targeted delivery or lymphatic orientation, liposomes can be administered in various routes, such as intravenous injection [110], oral administration [111], intraocular administration [112], pulmonary administration [113] and external use (including skin administration) [114] according to clinical needs. With the drug encapsulated in the lipid-like bilayer, the stability of the drug is enhanced by minimizing excretion and metabolism in a sustained release manner, which conduces to reduce the toxicity of the drug [115].

The PEG modification of the liposome surface can achieve extended residence time and long circulation by increasing the hydrophilicity of the microparticle surfaces and introducing steric hindrance to prevent opsonization and avoid being swallowed by phagocytes, known as the “stealth effect” [116,117]. This approach utilized the steric repulsion of PEG_2000_-DSPE to reduce the ORI-loaded liposomes uptake by RES and prolong blood circulation. Blood distribution was found to be significantly higher than in the heart after intravenous injection of ORI stealth liposomes to tumor-bearing mice. The MRT in the blood was extended to 35.92 h and the T_1/2_ to 24.24 h, compared with the ORI solution (MRT = 0.93 h, T_1/2_ = 24.89 h). The tumor inhibition rate of ORI stealth liposomes group was 85.4% at the tenth day, which was significantly higher than that of the ORI solution (63.7%) [118]. A similar result was observed in the report of Sun et al., where the lyophilized ORI-loaded PEGylated liposomes (ORI-PL) showed sustained release for three days, and anti-tumor activity was improved along with reduced ORI toxicity [119].

ORI-loaded FR-targeted liposomes (F-L-ORI) were obtained by conjugating folate to PEG_2000_-DSPE. The in vitro release showed burst and sustained two-phase with 95.1% accumulative release in 96 h. With a higher cell binding rate of F-L-ORI to HepG2, the IC_50_ value was reduced by 68% when compared with unconjugated L-ORI. The weight gain and HepG2 tumor volume reduction of the FL-ORI group were observed in mice with an inhibitory rate of 85.6% at the tenth day, which was significantly higher than the L-ORI (66.8%) and free ORI (40.8%) [120].

Guo et al. prepared a galactose-modified ORI-loaded liposome (NOH-ORI-LP) by the conjugation of *N*-octadecyl-4-((d-galactopyranosyl) oxy)-2,3,5,6-tetrahydroxy hexanamide (NOH) [121], which could be specifically recognized by ASGP-R. When the molar ratio of drug to lipid was 0.35, the EE of NOH-ORI-LP was improved to 94.1%. The pharmacokinetic behavior of NOH-ORI-LP fit the two-compartment model, exhibiting a relatively high plasma concentration for a long time compared with ORI solution. The Re of NOH-ORI-LP in the liver was as high as 4.28, indicating the liver-targeting ability of NOH-ORI-LP [122]. Table 4 summarized the research efforts have been devoted to ORI-loaded liposomes over the years.

### 3.6. Micelles

**Figure 8:**
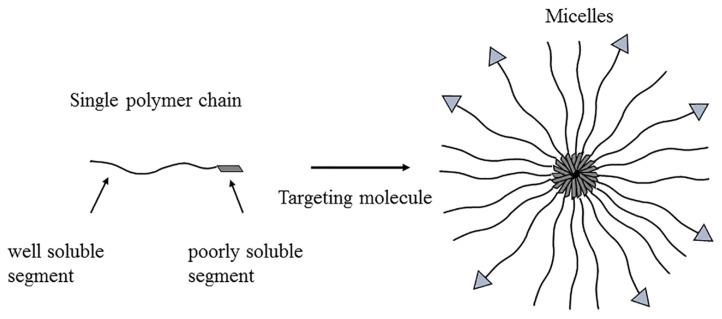
Illustration of copolymer micelle formation.

A micelle is a kind of self-assembled nanoparticle with a hydrophobic core and hydrophilic shell structure formed by amphiphilic molecular organization (Figure 8) [123,124]. The small size of the micelle enable it to avoid phagocytosis by the RES or absorption of tissues such as the liver and spleen, which improves the blood circulation of the drug in the blood and is beneficial for targeted delivery to specific tissues [125,126]. In addition, the materials used to prepare the copolymer micelles are generally degradable and can be eliminated by metabolism of the body to reduce allergic reactions [127].

There has been some research focusing on the ORI-M. Zhang et al. designed a block copolymer polyethylene glycol monomethyl ether polylactic acid (PEDLA) ORI-M, which showed a certain sustained release effect, while the EE and DL were not satisfactory [128]. The ORI-loaded monomethoxy poly (ethylene glycol)-poly(-caprolactone) (MPEG-PCL) micelles were readily resolved after freeze-drying and showed better transdermal properties than ORI solutions. MTT analysis results indicated that ORI-MPEG-PCL micelles significantly reduced the activity of mouse Lewis lung cancer LL2 cells [129]. Likewise, the IC_50_ value of ORI-loaded cholesterol formyl-chitosan (CF-CS) copolymer nano-micelles was about three times lower than copolymer nano-micelles and was about three times lower than that of the ORI solution on Hela cancer cells [130].

Consisting of multiple types of polymers, mixed micelles have a higher stability than singular ones, with a more uniform particle size distribution and core shell structure [131], which is favored for dissolution, sustained release and efficacy of drugs while inhibiting outflow [132,133]. Ke et al. prepared ORI-loaded nano-mixed micelles with Soluplus (SOL) and Pluronic P105 (P105). SOL with a lower critical micelle concentration combined with P105 to maximize the stability of the short length of poly (propylene oxide) chain of P105. The particle size was small (137.2 ± 1.65 nm) and the DL was 15.91%. It was confirmed that ORI-M remained stable after being diluted 200 times in artificial GI fluid [134]. Another study conjugated d-α-tocopheryl polyethylene glycol succinate TPGS and PLGA with disulfide bonds (TPGS-S-S-PLGA), and mixed the polymer with APDTKTQ-modified F68 to prepare an ORI-loaded mixed micelles, which could be specifically targeted to the receptor of advanced glycation end-products (RAGE). The APDTKTQ-RAGE affinity enhanced the cellular endocytosis of TSP-FP. The redox sensitivity of the S–S bond promoted the lysis of the carrier and the release of ORI by responding to the high concentration of intracellular glutathione, which further induced early apoptosis (78.2% with TSP-FP vs. 51.2% with free ORI) on BEL-7402 cells [135].

### 3.7. Combination of Formulations

Currently, the new materials have become an indispensable component of biomedical materials and drug controlled release systems [136]. Compared with conventional drug carriers, these materials are intelligent, environmentally friendly, versatile, stable, and usually reveal more preferable biocompatibility and larger DL, suggesting a new direction of formulations development when combined with conventional materials [137].

As an organic system, cubosomes provide a new method for building up new drug delivery systems. Shi et al. prepared ORI cubosomes with the phytantriol-propylene glycol-poloxamer 407-water system, which increased the solubility of ORI 5.2-fold with a unique internal three-dimensional double channel and lipid bilayer structure, which could maintain release for 24 h [138].

Inorganic systems, such as carbon nanotubes (CNTs), graphene, metal-organic frameworks (MOFs), gold nanocages (AuNCs) and selenium nanoparticles (Se NPs), have unique advantages as the drug vehicle, for example, a high Young modulus and large surface area. Embedded ORI in carbonyl-functionalized multi-walled CNTs (MWCNTs-COOH) by π-π stacking can reach a drug-loading of 82.6%. Through direct ingestion by the cell membrane, MWCNTs-ORI demonstrated an inhibition rate of 86.4% on HepG2 cells, which was significantly higher than the 39.2% of ORI-F [139]. 

Graphene oxide (GO) has good solubility and can be chemically modified for poorly soluble drug delivery due to its a large number of oxygen-containing reactive groups such as carboxyl groups, hydroxyl groups and epoxy groups [140]. Xu et al. attached a 6-arm PEG to GO and prepared GO-PEG ORI nanocarriers by loading ORI onto GO-PEG by the physical blending method. The DL ratio was as high as 105%, and the in vitro cytotoxicity results showed that the cell viability of MCF-7 cells treated with GO-PEG ORI for 48 h was 24% lower than that of ORI at a dose of 20 μmol/L [141]. Recently, Chai et al. prepared a universal nanoscale drug delivery system (GO-PEG_10K−6arm_) for extensive hydrophobic drugs with a particle size of about 200 nm, which has an EPR effect on tumor tissue to achieve passive targeting. The nanocomplexes (ORI@GO-PEG_10K−6arm_) obtained by combining ORI with PEG_10k−6arm_ have good thermostability and the DL is 10%. The cytotoxicity to cancer cells CAL 27, MG 63, HepG2 is approximately 10-times that of free ORI [142].

Composed of ions and terephthalic acid, Carboxylic acid iron MOFs overcome the shortcomings of low loading and poor stability of organic carriers, as well as the degradation difficulty of inorganic carriers. With adjustable pore size, good biocompatibility and a surface for modification, MOFs have been widely used as excellent nanocarriers in biomedical research [143]. For instance, Leng et al. prepared ORI@MIL-53 (Fe) with an MOF material, Materials of Institut Lavoisiser (MIL)-53 (Fe). The DL was up to 56.25 wt% with an in vitro release that lasted for more than seven days, and drug release in an alkaline environment was slower due to the pore size of MIL-53 (Fe). The cytotoxicity study showed that the anticancer rate of ORI@MIL-53(Fe) was 90.62% on HepG2 cells, suggesting its potential for anti-cancer applications [144]. Chen et al. prepared ORI@MOF-5 based on the carrier of nano-MOF-5. It demonstrated a significant cytotoxicity on HepG2 cells (IC_50_ = 22.99 μg/mL) and sustained release was not affected by pH [145]. As a result, MOFs have the potential to be a drug carrier material.

**Figure 9:**
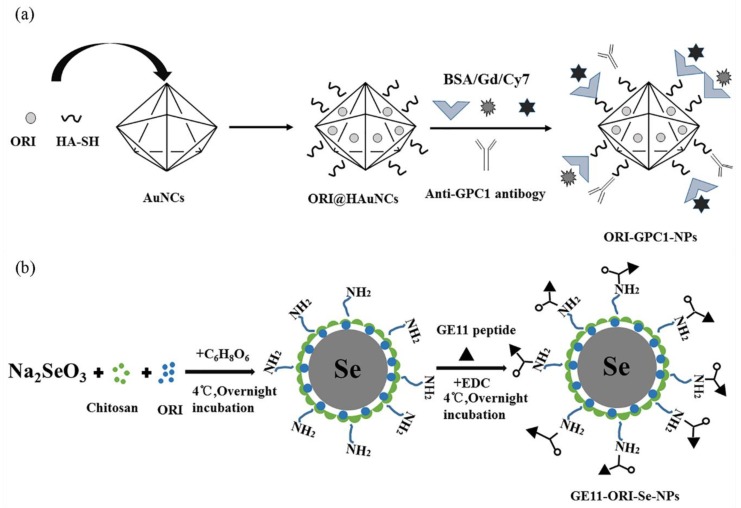
Schematic diagram of preparation of (**a**) ORI-GPC1-NPs. (**b**) GE11-ORI-Se NPs.

Targeted delivery of ORI can be achieved with inorganic carriers when modified by the ligand. Qiu et.al. constructed an ORI-loaded multifunctional theoranostic nanoplatform (ORI-GPC1-NPs) composed of hyaluronic acid (HA)-modified AuNCs, anti-Glypican-1 antibody, ORI, gadolinium (Gd), and Cy7 probe (Figure 9a). The hollow cage inside AuNCs increased the DL. HA, with high stability and good biocompatibility, worked as an ideal surface-coating of AuNCs for anti-GPC1 antibody conjugation, which effectively enhanced targeting to GCP1 overexpression pancreatic cancer cells and antitumor activity at lower ORI concentrations. The NIRF and MRI imaging of loaded Cy7 probes could be utilized for an early diagnosis and in vivo biodistribution study. This versatile nano-platform is expected to become a potential approach for precise theoranostics [146].

Se NPs deliver drugs to cancer cells by conjunction with targeting biomolecules. GE11 is a highly efficient EGFR-targeting peptide. With GE11 conjugated to the Se NPs on the CS coating, the GE11-ORI-Se NP was designed (Figure 9b) for treatment of esophageal laryngeal carcinoma (ESCC). After specific endocytosis into the KYSE-150 cells, the ORI was released in the acidic lysosomes due to the pH-dependent characteristics of CS. It was observed that the expression of CD31, which was related to the angiogenesis of ESCC, was downregulated along with the activation of immune system [147]. Another example is ORI@ GE11-GO, of which GE11 peptide were conjugated with GO. The study showed that ORI@ GE11-GO effectively induced apoptosis of esophageal cancer cells (KYSE-30 and EC109 cells) by activating mitochondrial-dependent apoptosis [148].

### 3.8. Others

In addition to the dosages mentioned above, techniques such as solid dispersions, nanogels and self-microemulsifying drug delivery systems (SMEDDS) have also been investigated in the ORI delivery system field. Li et al. prepared an ORI solid dispersion using CO_2_ as the anti-solvent and a hydrophilic polymer polyvinylpyrrolidone K17 (PVP K17) as the drug carrier by the gas anti-solvent technique, obtaining smaller particles without organic solvents in one-step granulation. Pharmacokinetic results showed that the bioavailability and of the ORI solid dispersion increased by 26.4 times compared with the physical mixture of ORI and PVP K17 [149]. An ORI-loaded nanogel was explored with copolymers, chitosan-graft-poly(*N*-isopropylacrylamide) (CS-g-PNIPAm), whose in vitro release showed pH-dependent profiles and high anti-cancer activity was achieved at pH = 6.5 [150]. Following modifications of nanogels with galactose, ORI uptake was enhanced in HepG2 cells via galactose-specific receptor-mediated endocytosis [151]. It was found that the higher antitumor activity could be achieved with a larger degree of galactose substitution. At the material ratio of Maisine 35-1 and Labrafac CC (1:1): Cremopher El: Transcutol *p* = 30%:46.7%:23.3%, the highly stable and stable ORI SMEDDS could be formed [152], with bioavailability 2.2-times higher than ORI suspension. Nevertheless, the drug was completely released 12 h after administration.

## 4. Conclusions

As a natural active ingredient, ORI has a broad prospect of development with its wide range of pharmacological activities. At present, ORI is the main active ingredient of *Rabdosia rubescens*, which is an OTC herbal medicine (“Donglingcao Pian”) approved by NMPA. However, it is rare to be used as a monomeric drug in clinical applications, considering its limited solubility and bioavailability.

In recent years, many ways have been investigated to improve the solubility and bioavailability of ORI, such as optimizing the chemical structure, loading ORI into nanoparticles, liposomes, etc. Nevertheless, there remains several issues and new directions for future development: (1) In the field of pharmaceutical chemistry, chemical structure modification to improve the water solubility of drugs is the basic method to consider and solve the problem from the essence of dissolution. Based on the structural characteristics of ORI, its structural modification is currently concentrated in four directions: The derivatization of hydroxyl groups, modification of A-ring, modification of the enone system, and the transformation and derivatization of the framework structure. Salt formation and polar group introduction are the basic strategies to improve water solubility, and moderation of the covalent warhead reactivities at the D-ring may be a viable approach to the discovery and development of effective and druglike ORI derivatives in the future. (2) Though some structural modifications can improve membrane permeability and solubility of ORI, the structure–activity relationship was rarely studied and the target and molecular mechanism of action were not clear enough; some target compounds lacked further activity verification. From some studies, the target or regulated signaling pathway of ORI is not single, which suggests that our future research should be based on multiple disease-related targets and signaling pathway networks. (3) The EE and DL of the ORI-loaded carriers are not high enough, requiring larger amounts of carriers in use. However, some carrier materials are not completely degradable and accumulate in the human body after long-term use, which leads to side effects such as liver and kidney toxicity and vascular inflammation. It is worth noting that cyclodextrin mucous membrane can lead to renal toxicity; polyoxyethylene castor oil or polysorbate 80, the surfactant used for injection, results in neurotoxicity and severe allergic reactions; and the improper application of phospholipids will give rise to a hemolytic effect. A safe and effective carrier material is the key to develop the drug delivery system. (4) Despite the fact that cyclodextrin inclusion, nanoparticle, liposome, micellar, and solid dispersion improve the solubility and bioavailability of ORI, they face some other challenges: Liposomes have stability problems like aggregation, fusion and drug leakage during storage; preparations of some nanoparticles, and polymer micelles were still limited in the laboratory scale; the in vivo environment is too complicated for a targeting effect; the mechanism of action for some materials is not clear. Currently, there is no active targeted formulation of ORI in the market. The preparation of drug delivery dosage forms for intravenous administration is an effective strategy to improve the pharmacokinetic properties of ORI derivatives considering bioavailability.

In summary, with the present achievements, the strategy of structural modification has great potential to improve the solubility, bioactivity and safety of ORI, while pharmaceutical formulation has a good effect in improving solubility and bioavailability. Both of the strategies are the research directions of ORI in the future. The combination of the derivatization method and preparation method can achieve better results in future research, with high efficiency, low toxicity and good stability.

## Figures and Tables

**Table 1:** Pharmacological activities of ORI.

Activities	Mechanism of Actions	Ref.
Anti-cancer	Repressing cell cycle, down-regulating telomerase activity, inhibiting cell membrane sodium pump activity, inducing tumor cell apoptosis	[3,4]
Anti-bacterial	-	[5]
Neuroregulation	Upregulating the production of the neurotrophic factor and nerve growth factor	[6]
Anti-oxidation	Scavenging active oxygen free radicals	[7]
Depressurization	Inducing pulmonary artery smooth muscle cell apoptosis	[8]
Anti-inflammation	Down-regulating the inflammatory factors IL-figure1β, IL-6, and IL−33; Blocking the interaction between NLRP3 and NEK7, inhibiting NLRP3 inflammasome activation	[9,10,11]
Immune-modulating	Promoting CD4+ /CD25+ Treg differentiation; modulating Th1/Th2 balance	[12]
Analgesia	Reducing colonic EC cell hyperplasia and 5-HT availability	[13]

**Table 2:** Cytotoxicity and pharmacodynamics study of ORI nanosuspension.

Research Objects	Biological Activity or Mechanism	Ref.
New Zealand white rabbits, Kunming strain mice	Particle size affects pharmacokinetics and tissue distribution	[68]
K562 cell, S-180 tumor-burdened mice	Significantly more toxic to K562 cells than free ORI (*p* < 0.01)	[69]
PC-3 cell	Inducing early apoptosis and enhancing growth suppression	[70]
SMMC-7721 cell, H22 tumor-bear mice	Arresting cells in the G2/M phase, tumor volume response ORI-N has a higher anti-tumor effect	[71]
MCF-7 cell	Reducing the expression of Bcl-2 and increasing Bax	[72]
PANC-1 cell	Suppressing the expression of B1 and p-cdc2 (T161) on G2/M cell phase	[73]
SD-rats	Improving dissolution and permeability by interaction with absorptive epithelia and anti-drug efflux.	[74]

SD = Sprague Dawley.

**Table 3:** Examples of different ORI polymer nanoparticles.

Delivery Systems	Administration	Results	Ref.
ORI-PLA-NPs	Intravenous administration in mice at a dose of 25 mg/kg	High concentration of ORI in liver, lung and spleen	[78]
ORI-PCL-PEO-PCL-NPs	Intravenous administration in H22 tumor-bear mice at a dose of 8 mg/kg	Tumor volume and weight decreased, and the survival rate increased to 169.6%	[80,81]
ORI-PLA-RGD-NPs	Intravenous administration in the H22 tumor-bear mice at a dose equivalent to 20 mg/kg of ORI	Enhanced targeting effect; tumor volume and weight were significantly reduced (*p* < 0.01), with average survival length extended from 27 days to 41 days compared with ORI-PLA-NP	[82]
ORI-GC-NPs	Intravenous administration in mice at a dose of 1 mg/kg	Prolonging MRT_0–t_ from 3.415 h to 14.042 h, liver *AUC*_0–t_ increasing by 6.4-fold compared with ORI solution	[84]
ORI-GB-NPs	Intravenous administration on Wistar rats at a dose of 14 mg/kg and Kunming strain mice at 20 mg/kg	The retarded in vitro release with increasing of crosslinking agent glutaraldehyde; liver active targeting; enhancing the drug plasma concentration and prolonging the circulation time	[85,86]
ORI-Gal-PT-NPs	Treating HepG2 cells at concentrations of 5.0, 7.5, 10 and 12.5 mg/mL	The apoptosis increasing by 4−5.6% compared with ORI solution	[87]

MRT = Mean Retention time.

**Table 4:** Examples of ORI-loaded liposomes.

Materials	Preparation Methods	Administration	Results	Ref.
Cholesterol and soy lecithin, CPA, PEG_2000_-DSPE	Thin-film ultrasonic dispersion method	Mice injected via the tail vein at 33.5 mg/kg	Enhancing T_1/2_ from 0.65 h to 24.89 h, 85.4% tumor inhibition rate	[118]
PEG	Ethanol injection method	H22 tumor-bearing mice	Drug accumulation in tumor cells, prolonged plasma residence	[119]
CH, soy bean lecithin, folic acid, *N*,*N*’-dicyclohexyl carbodiimide, PEG_2000_-DSPE	Thin-film ultrasonic dispersion method	Intravenous administration in tumor-bearing nude BALB/_C_ mice at a dose of 1.5 × 10^−2^ g/kg/day	Body weight gain, 85.6% tumor inhibition	[120]
EPC, CH, NOH,	Ethanol injection method	Intravenous administration in mice at a dose of 30 mg/kg	Enhancing MRT_0–t_ from 1.69 h to 9.40 h, Enhancing *AUC*_0–t_ by 4.58-fold, 61.18% Te	[121]

T_1/2_ = Half-Life; Te = Targeting Efficiency; CPA= Cyclophosphamide; PEG_2000_-DSPE= Polyethylene Glycoldistearoylphosphatidyleth-anolamine; CH = Cholesterol; EPC = Egg Phosphatidylcholine; NOH = *N*-octadecyl-4-((d-galactopyranosyl) oxy)-2,3,5,6-tetrahydroxy Hexanamide.
